# Ciliary neurotrophic factor stimulates cardioprotection and the proliferative activity in the adult zebrafish heart

**DOI:** 10.1038/s41536-019-0064-9

**Published:** 2019-01-24

**Authors:** Thomas Bise, Anne-Sophie de Preux Charles, Anna Jaźwińska

**Affiliations:** Department of Biology, University of Fribourg, Chemin du Musée 10, 1700 Fribourg, Switzerland

## Abstract

Unlike mammals, adult zebrafish can regenerate their hearts after injury via proliferation of cardiomyocytes. The cell-cycle entry of zebrafish cardiac cells can also be stimulated through preconditioning by thoracotomy, a chest incision without myocardial damage. To identify effector genes of heart preconditioning, we performed transcriptome analysis of ventricles from thoracotomized zebrafish. This intervention led to enrichment of cardioprotective factors, epithelial-to-mesenchymal transition genes, matrix proteins and components of LIFR/gp130 signaling. We identified that inhibition of the downstream signal transducer of the LIFR/gp130 pathway through treatment with Ruxolitinib, a specific JAK1/2 antagonist, suppressed the cellular effects of preconditioning. Activation of LIFR/gp130 signaling by a single injection of the ligand Cilliary Neurotrophic Factor, CNTF, was sufficient to trigger cardiomyocyte proliferation in the intact heart. In addition, CNTF induced other pro-regenerative processes, including expression of cardioprotective genes, activation of the epicardium, enhanced intramyocardial Collagen XII deposition and leucocyte recruitment. These effects were abrogated by the concomitant inhibition of the JAK/STAT activity. Mutation of the *cntf* gene suppressed the proliferative response of cardiomyocytes after thoracotomy. In the regenerating zebrafish heart, CNTF injection prior to ventricular cryoinjury improved the initiation of regeneration via reduced cell apoptosis and boosted cardiomyocyte proliferation. Our findings reveal the molecular effectors of preconditioning and demonstrate that exogenous CNTF exerts beneficial regenerative effects by rendering the heart more resilient to injury and efficient in activation of the proliferative programs.

## Introduction

In adult mammals, a damaged myocardium cannot be restored because cardiomyocytes are not sufficiently proliferative.^1,2^ By contrast, zebrafish cardiac cells can activate the morphogenetic programs and enter the cell cycle to regenerate an injured ventricle.^3–9^ Lineage tracing analyses have demonstrated that the new myocardium originates from remaining cardiomyocytes (CMs) at the site of injury.^10–13^ The physiological growth of juvenile and adult fish also involves CM proliferation, however, without a noticeable activation of injury-responsive programs.^14,15^ Non-cardiac tissues of the heart, such as epicardium, endocardium, vasculature, fibroblasts, nerves and immune cells, provide an environment for stimulation of cardiac cells.^16–28^

Our laboratory has recently demonstrated that chest incision or intraperitoneal injection of immunogenic particles can induce the cell-cycle entry of CMs.^29^ These interventions also resulted in an upregulation of cardioprotective genes, such as *txn, cxcl12a, hmoxla and hsp5a*. As opposed to regeneration, no CM dedifferentiation was observed after thoracotomy, indicating a different form of cardiogenesis in both contexts. Importantly, thoracotomy at a few days before ventricular cryoinjury increased cell survival and enhanced cell proliferation during the first week of regeneration.^29,30^ Based on these beneficial effects, we proposed that the surgical opening of the pericardium provides a model of cardiac preconditioning in zebrafish. Thus, the cell-cycle entry of CMs can be enhanced by a preconditioning procedure in zebrafish.

Preconditioning is a systemic self-defense mechanism that is invoked by exposure to low doses of a harmful stimulus and enables tissues to better withstand the deleterious effects of subsequent more severe injuries.^31,32^ In mammals, the target organs of preconditioning include the heart, brain, kidney, liver and skeletal muscle, all of which temporarily become more resilient to damage after a small injury.^33^ A variety of remote stimuli can elicit organ protection, such as electroacupuncture, nociceptor activation through capsaicin and surgical skin incision. In clinical trials, cardiac preconditioning strategies rely on non-invasive remote insults, such as cycles of inflation–deflation using a blood pressure cuff on the patient’s arm.^34–36^ Studies in rodents revealed that preconditioning is associated with an elevated expression of pro-angiogenic, antioxidant and cytoprotective genes.^31,37^ From an evolutionary perspective, preconditioning can be considered an adaptive trait that increases the fitness of organisms by strengthening and preparing the body to cope with environmental hazards.^38,39^ The molecular mechanisms of cardiac preconditioning in the zebrafish model have not yet been investigated.

In this study, we used thoracotomy as a model of cardiac preconditioning in zebrafish. To identify molecular factors involved in this process, we compared transcriptional profiles of ventricles from intact and thoracotomized animals. This analysis revealed upregulation of cardioprotective factors, epithelial-mesenchymal transition (EMT) genes and extracellular matrix (ECM) components. Among signaling pathways, we found that multiple members of the LIFR/gp130 (Leukemia inhibitory factor receptor / Glycoprotein 130) cascade displayed a remarkable expression change at one day after thoracotomy. LIFR and gp130 are structurally related and ubiquitously expressed receptors that form a heterodimeric signaling complex at the cell surface through binding to cytokines of the IL (Interleukin)-6-type family.^40^ Activation of the receptor complex leads to phosphorylation of JAKs (Janus tyrosine kinases), resulting in phosphorylation of STATs (Signal transducers and activators of transcription), which translocate into the nucleus and initiate gene expression. The activity of the gp130 receptor is negatively regulated by the feedback inhibitors SOCSs (Suppressors of cytokine signaling). LIFR/gp130 signaling can trigger a wide range of effects depending on the cell type and the cellular state.^41–43^

Here we investigated whether inhibition of LIFR/gp130 signaling by a pharmacological selective inhibitor of the JAK1/2 activity, Ruxolitinib, abrogates cardiac preconditioning after thoracotomy. Furthermore, to test whether activation of LIFR/gp130 is sufficient to elicit the preconditioned phenotype, we synthetized zebrafish CNTF (zCNTF), a LIFR/gp130 ligand,^40^ and injected it into the pericardial cavity. To determine if the *cntf* gene is necessary for preconditioning-induced cardiomyocyte proliferation, we generated mutant zebrafish and analyzed their hearts after thoracotomy. Our findings demonstrate that *cntf* regulates several effects of cardiac preconditioning in zebrafish.

## Results

### Transcriptional changes after thoracotomy suggest the activation of cytoprotection

**Fig. 1:**
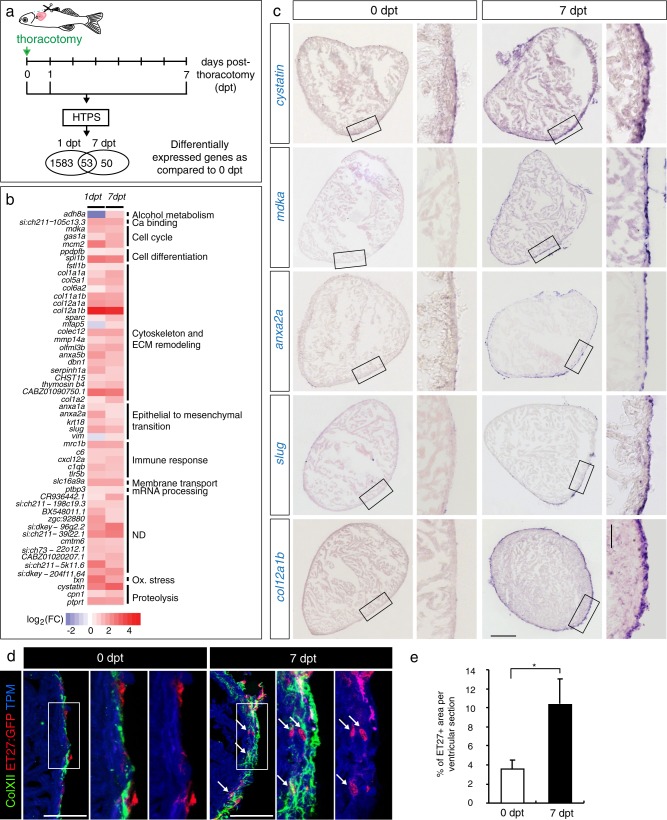
Transcriptional changes after thoracotomy suggest the activation of cardioprotective and EMT genes in the epicardium. **a** Experimental design. High throughput sequencing (HTPS) of ventricles collected at 0 (control), 1 and 7 days post-thoracotomy (dpt). For each group, RNA was extracted from a pool of 8 ventricles. The encircled numbers indicate differentially expressed genes at each time point. The middle number depicts differentially expressed genes common for both time points. **b** Heat-map representation of the common 53 differentially expressed genes at 1 and 7 dpt. Genes are grouped according to biological function. Fold changes are represented in log2 scale (blue: log2 < 0; red: log2 > 0). **c** *In-situ* hybridization of ventricular transversal sections reveals upregulation of several candidate genes (purple) in the epicardial and sub-epicardial region at 7 dpt, compared to control hearts at 0 dpt. The frames indicate the part of the section that is magnified on the right side of each image. *n* ≥ 3 hearts. Scale bar for the whole section, 100 μm; for the magnified area, 50 μm. **d** Immunofluorescence staining of ventricular sections of transgenic fish *ET27:EGFP* (red) with antibodies against cardiac Tropomyosin (TMP, blue) and ColXII (green). At 0 dpt, ET27:EGFP + cells and ColXII are confined to the epicardium. At 7 dpt, *ET27:EGFP* + cells and ColXII expand and infiltrate the myocardium. Arrows indicate intramyocardial *ET27:EGFP* + cells. Scale bar, 50 μm. **e** Quantification of intramyocardial *ET27:EGFP* + area per ventricular section area. Superficial epicardial ET27 + cells were not included in measurements. *n* ≥ 3 hearts, 3 sections per heart each. *P < 0.05 with student’s t-test. Error bars represent standard error of the mean (s.e.m.) (This applies to the all subsequent figures)

Thoracotomy induces preconditioning in the zebrafish heart, but the molecular pathways mediating cardioprotection and proliferation remain unknown.^29^ To identify biological processes activated in the heart by thoracotomy, we performed transcriptome high throughput sequencing. Expression profiles of ventricles from uninjured animals were compared to those from fish at 1 day post-thoracotomy (dpt), the early phase after stimulation, and at 7 dpt, when an advanced preconditioning effect should be detected (Fig. 1a). We identified 1638 and 103 differentially expressed genes at 1 and 7 dpt, respectively, compared to uninjured ventricles at 0 dpt (Suppl. Data 1; Suppl. Fig. S1). To identify the effects of thoracotomy, we first focused our analysis on 53 common genes at 1 and 7 dpt, which we manually annotated (Fig. 1b; Suppl. Data 2). In mammals, one of the key features of preconditioning is the induction of cell protection programs.^37^ Consistently, the expression of several orthologs of mammalian genes associated with cytoprotection was increased, namely a pleiotropic cytokine *midkine a* (*mdka*),^44,45^ *chemokine C-X-C motif ligand 12a* (*cxcl12a*, also known as *sdf-1α*),^46^ an oxidative stress factor *thioredoxin* (*txn)*,^47^ a glycoprotein *cystatin*,^48^ a carnitine efflux transporter *slc16a9*,^49^ a short peptide *thymosin β4*,^50^ a plasma metalloprotease *carboxypeptidase N1*,^51^ and an alcohol dehydrogenase *adh8a*^52^ (Fig. 1b, Suppl. Data 2). This finding suggests that the molecular players of preconditioning are conserved between mammals and fish.

We selected several common candidate genes with the highest expression change for further analysis by *in-situ* hybridization at 7 dpt. We found that most of the genes were upregulated at the surface of the ventricle after thoracotomy (Fig. 1c). Beside cardioprotective genes, we identified a few mediators of epithelial-to-mesenchymal transition (EMT), such as *annexins* and *slug* (Fig. 1c, Suppl. Data 2). Among extracellular matrix (ECM) components, we found enrichment of several collagens, particularly of two paralogous genes encoding *col12a1a* and *col12a1b*, which belong to the group of fibril-associated collagens with interrupted triple helical domains (FACIT).^53^ ColXII proteins do not assemble into rigid fibrils, but form flexible bridges between matrix fibers.^54^ The upregulation of several EMT and FACIT genes on the heart surface indicates that activation of epicardial cells might be an important mechanism of preconditioning after thoracotomy.

To further investigate this observation, we used the transgenic fish strain *ET27:EGFP*, which demarcates the epicardium,^55^ and performed immunofluorescence staining against ColXII. In uninjured control fish, as previously shown,^19,56^ *ET27:EGFP*-expressing cells and ColXII-positive fibrils were mostly confined to the superficial layer of the heart (Fig. 1d). Interestingly, at 7 dpt, we found a 3-fold increase of *ET27:EGFP*-expressing cells entirely within the underlying myocardium and an extensive infiltration of ColXII (Fig. 1d, e). Our findings suggest that thoracotomy triggers expansion of epicardial cells and ColXII in the ventricle.

### The LIFR/GP130 pathway is activated at 1 day after thoracotomy

Our next aim was to use the high throughput sequencing data to identify a signaling pathway that can initiate cardiac preconditioning at 1 dpt. An enrichment analysis of pathway maps revealed a significant representation of genes involved in the signaling of interleukin-6 (IL-6) family cytokines.^40^ In our dataset, the expression of *leukemia inhibitory factor receptor alpha b* (*lifrb*, also known as *CD118*) and *glycoprotein-130* (*gp130*), also known as the *interleukin-6 signal transducer (il6st)*, and several of their downstream effectors (*jak1, stat1b, stat3, mapk12a, junba, cebpb*) or targets (*mmp13a, timp2b, ldlr, ldlrad3, socs3b*) were modulated at 1 dpt (Suppl. Fig. S2a, Suppl. Data 3). Transcripts of *socs3b, mmp13a, timp2b* were detected on the outer layer of the heart, as determined by *in-situ* hybridization (Suppl. Fig. S2b). We concluded that the LIFR/gp130 pathway is upregulated in the epicardium after thoracotomy.

In mammals, LIFR and GP130 receptors act as heterodimers, which are activated by IL-6 type ligands, such as leukemia inhibitory factor (LIF), oncostatin M (OSM), ciliary neurotrophic factor (CNTF), cardiotrophin-1 (CT-1) and cardiotrophin-like factor (CLCF).^40^ To investigate the role of the LIFR/GP130 pathway in cardiac preconditioning, we first aimed to determine which of the LIFR-binding cytokine displays the highest expression in the zebrafish heart at 1 dpt. No zebrafish orthologs were identified for *osm* and *ct-1*, but *cntf, clcf1* and *lif* genes have been annotated in the *Danio rerio* genome (Ensemble, release 89). qRT-PCR analysis revealed that among these cytokines, only *cntf* displayed a significant upregulation at 1 dpt (Suppl. Fig. S2c). Consistently, *in-situ* hybridization detected *cntf* transcripts in the outer layer of the preconditioned ventricle (Suppl. Fig. S2d). These data suggest that the LIFR/gp130 signaling pathway might be involved in cardiac preconditioning.

### Enhanced proliferation and ColXII deposition observed after thoracotomy are dependent on the JAK/STAT pathway

**Fig. 2:**
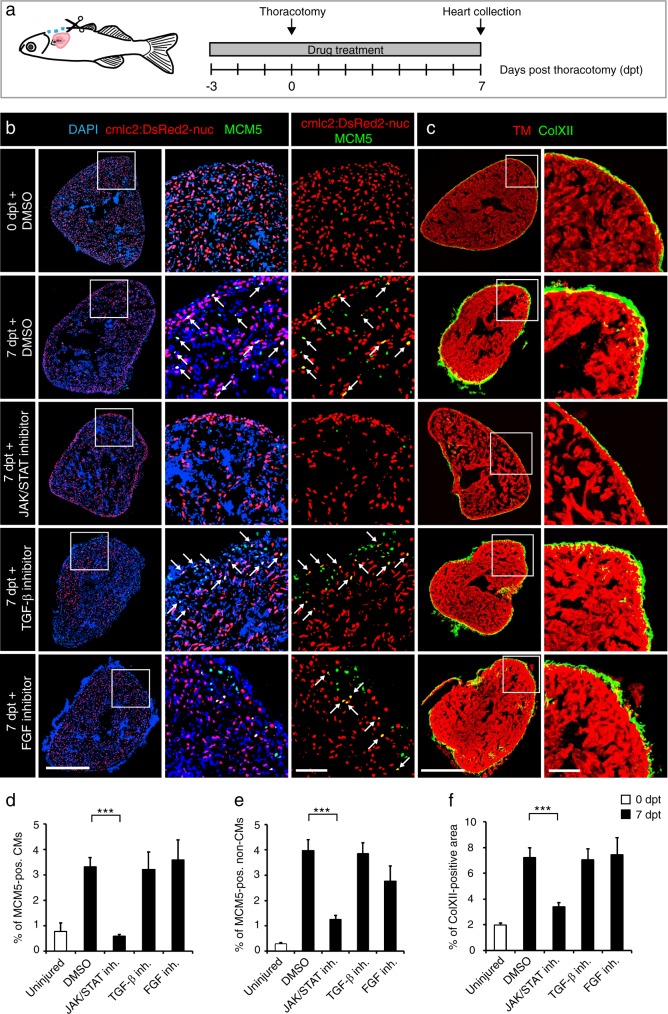
The cell-cycle entry and the deposition of ColXII after thoracotomy are dependent on the JAK/STAT3 pathway. **a** Experimental design. The requirement of different pathways for the increased CM mitotic activity and the ColXII deposition observed after preconditioning was tested at 7 dpt by using specific inhibitors of JAK/STAT (1 µM Ruxolitinib), TGF-β (20 µM SB431542) or FGF (10 µM PD173074) signaling. **b**, **c** Transversal sections of hearts treated with different drugs indicated at the left side. Scale bar for the whole section, 500 μm; for the magnified area, 100 μm. **b** Ventricle of transgenic fish *cmlc2:DsRed2*-nuc (red) immunostained for the G1/S-phase marker MCM5 (green). Some double positive cells are indicated with arrows. Treatment with Ruxolitinib markedly reduces cells proliferation in the ventricle. **c** Ventricle of wild type fish immunostained against Tropomyosin (red) and ColXII (green). In the presence of Ruxolitinib, intramyocardial ColXII is reduced. **d** Proportion of MCM5 + nuclei among *cmlc2:DsRed2-nuc* + nuclei. **e** Proportion of MCM5 + *cmlc2:DsRed2*-nuc-negative nuclei among DAPI + nuclei. **f** Proportion of the ColXII-positive area within the surface of ventricular section. *n* ≥ 4 hearts; ≥ 2 sections per heart; ***P < 0.001

Among upregulated genes at 1 dpt, we identified *socs3b*. Expression of *socs3* genes is considered a sensitive readout of JAK/STAT3 activation.^57^ To test the importance of the LIFR/gp130 pathway in cardiac preconditioning, we disrupted its downstream JAK/STAT3 signaling using Ruxolitinib (INCB018424), which selectively inhibits JAK1/2 activity in mammals, and has been validated in zebrafish.^58^ We designed an experiment to assess the effects of the drug on cell proliferation and ColXII deposition at 7 dpt (Fig. 2a). To distinguish between cardiac and non-cardiac cells, we used *cmlc2:DsRed2-nuc* transgenic fish that express a red fluorescent protein in the nuclei of CMs.^59^ To detect the proliferative activity, we applied MCM5 as a marker of the G1/S phase in the cell cycle.^60^ Consistent with our previous study,^29^ at 7 dpt, the preconditioned hearts contained 4- and 10-times more MCM5-positive cardiac and non-cardiac nuclei, respectively, than uninjured control hearts (Fig. 2b, d, e). This enhanced proliferative activity was suppressed by the treatment with the JAK/STAT3 inhibitor, Ruxolitinib (Fig. 2b, d, e). To verify the specificity of this phenotype, we suppressed two other signaling pathways that are essential for heart regeneration, namely TGF-β and FGF using their specific antagonists, SB431542 and PD173074, respectively.^20,61^ Interestingly, the inhibition of TGF-β and FGF signaling did not influence cell proliferation after thoracotomy (Fig. 2b, d, e). We concluded that the stimulation of the cell cycle in the preconditioned hearts might be dependent on JAK/STAT3 signaling.

To determine the role of JAK/STAT3 signaling on modifications of the extracellular matrix, we assessed deposition of ColXII. At 7 dpt, the amount of ColXII-positive fibers displayed a 4-fold increase compared to uninjured control (Fig. 2c, f). Inhibition of the JAK/STAT3 pathway resulted in a decrease of ColXII accumulation, an effect that was not caused by the suppression of TGF-β and FGF signaling (Fig. 2c, f). Taken together, our observations suggest that the activation of JAK/STAT3 signaling promotes cell proliferation and ECM reorganization in intact preconditioned hearts. Furthermore, the roles of TGF-β and FGF might be restricted to the regenerative processes, thereby reemphasizing the unique role of JAK/STAT3 during heart preconditioning.

### Injection of zCNTF results in rapid upregulation of preconditioning genes

In mammals, CNTF is not essential for development and survival, but it can elicit powerful neuroprotective and metabolic effects, when ectopically administrated.^62^ As *cntf* transcripts were detected on the heart surface after thoracotomy (Suppl. Fig. S2c, d), we asked whether CNTF is a relevant activator of LIFR/GP130 signaling in the context of cardiac preconditioning. Considering that the mammalian CNTF recombinant protein only displays an approximately 20% identity with the zebrafish ortholog, we synthetized the zebrafish CNTF protein for our functional study (Suppl. Fig. S3).

To test the effects of exogenous zCNTF on the heart, we injected 2.5 μl solution containing 250 ng of this protein into the pericardial cavity, whereby the amount of protein was chosen based on previous studies in zebrafish^63^ and rodents.^62^ For the control, we used the same quantity of human immunoglobulins (hIgG), which we selected as unspecific proteins with a neutral pharmacological activity, as verified in vivo.^64^ To determine if zCNTF injection alters gene expression in a similar pattern as thoracotomy, we performed *in-situ* hybridization with the validated probes (Suppl. Fig. S4a). We found that several LIFR/GP130 downstream factors, such as *socs3b, mmp13a*, *timp2b*, as well as the cardioprotective gene *cystatin* and the EMT-factor *anxa2a*, were upregulated at 1 day post-injection (dpi), as compared to hIgG control (Suppl. Fig. S4b). Furthermore, zCNTF injection into *ET27:EGFP* fish stimulated invasion of epicardial cells into the myocardium in a similar manner as thoracotomy, as assessed at 7 dpi (Suppl. Fig. S4c, d). Thus, delivery of exogenous zCNTF is sufficient to induce expression of preconditioning genes and to activate the epicardium in the intact heart.

### Exogenous zCNTF is sufficient to increase myocardial mitotic activity, ColXII deposition and leucocyte recruitment to uninjured heart

**Fig. 3:**
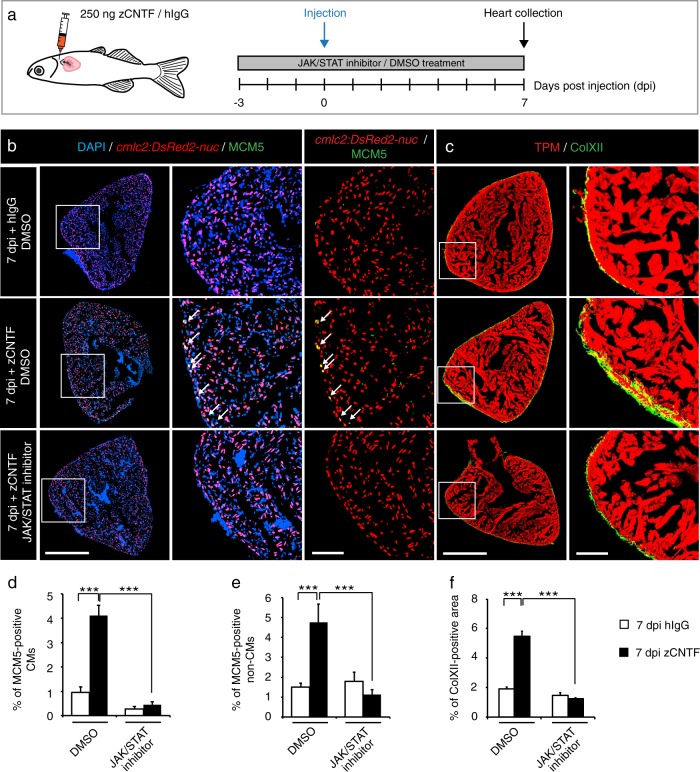
Single injection of zCNTF elevates the mitotic activity and ColXII deposition. **a** Experimental design to assess effects of a single intrathoracic injection of zCNTF in the presence of the JAK/STAT inhibitor, 1 µM Ruxolitinib, at 7 dpi. **b**, **c** Immunofluorescence staining of heart sections. Scale bar for the whole section, 500 μm; for the magnified area, 100 μm. **b** Transversal heart sections of transgenic fish *cmlc2:DsRed2-nuc* (red) to demarcate CM nuclei, immunostained with the G1/S-phase marker MCM5 (green) display enhanced CM proliferation (arrows) after zCNTF injection in the absence of the JAK/STAT inhibitor. **c** Transversal heart sections of wild type fish immunostained with of ColXII (green) and Tropomyosin (red). Intramyocardial ColXII is increased in zCNTF-injected hearts. JAK/STAT inhibition abolished this effect. **d** Proportion of MCM5-positive cells among *cmlc2:DsRed2-nuc*/DAPI-positive CM nuclei. **e** Proportion of MCM5 + *cmlc2:DsRed2-nuc*-negative nuclei among DAPI + nuclei. **f** Proportion of the ColXII-positive area within the surface of ventricular section. *n* ≥ 4 hearts; ***P < 0.001

To test whether zCNTF is sufficient to exert preconditioning effects similar to thoracotomy, we performed intrathoracic injections into *cmlc2:DsRed2-nuc* transgenic fish in the presence of Ruxolitinib or control treatments (Fig. 3a). Remarkably, a single injection of zCNTF was sufficient to increase the number of MCM5-expressing CMs by nearly 4-fold, compared to hIgG-injected fish (Fig. 3b, d, e). Furthermore, we observed a similar increase of mitosis, using phospho-Histon H3 immunostaining (Suppl. Fig. S5). zCNTF also induced a 3-fold increase in ColXII deposition (Fig. 3c, f). All these effects were abolished by the inhibition of JAK/STAT3 signaling with Ruxolitinib (Fig. 3b–f). Thus, zCNTF stimulates the cell cycle entry of CMs and ECM remodeling in intact zebrafish hearts by activating the JAK/STAT3 pathway.

**Fig. 4:**
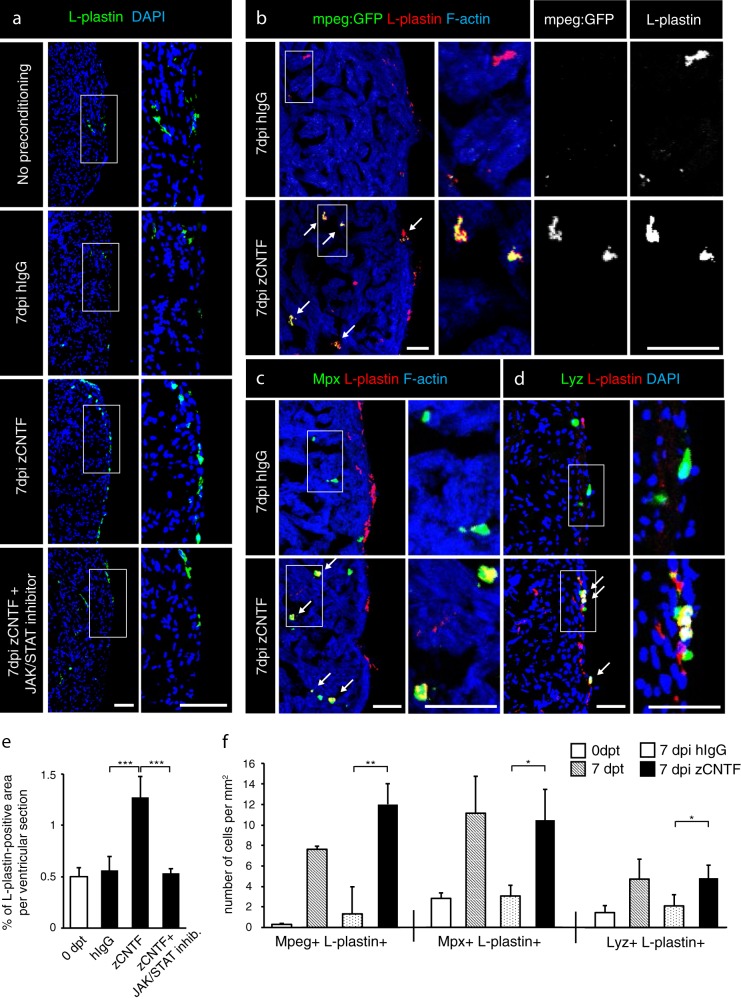
Exogenous zCNTF recruits leucocytes into the zebrafish uninjured heart. **a**–**d** Immunofluorescence staining of heart sections from different conditions as indicated at the left side of the images. **a** Hearts of wild type fish stained for L-plastin (green) and DAPI (blue). Single injection of zCNTF results in higher accumulation of L-plastin in the heart at 7 dpi. This effect is abolished in the presence of the JAK/STAT inhibitor, 1 µM Ruxolitinib. Scale bars, 100 μm. **b** Hearts of transgenic fish *mpeg1:EGFP* (green) stained for L-plastin (red) and F-actin (Phalloidin, blue). The number of *mpeg1:EGFP*/L-plastin-expressing cells is increased at 7 days after zCNTF injection. Some double positive cells are indicated with arrows. Scale bers 50 μm. **c**, **d** Ventricles of wild type fish stained for Mpx (c, green) or Lyz (d; green) and F-actin (Phalloidin, blue). The number of double positive leucocytes is increased at 7 days after zCNTF injection (arrows). Scale bars, 50 μm. **e** Quantification of L-plastin + area in ventricular sections. **f** Quantification of cells expressing *mpeg1:EGFP*, Mpx and Lyz normalized to the ventricle area at different conditions. *n* ≥ 4 hearts; ≥ 2 sections per heart; *P < 0.05; **P < 0.01; ***P < 0.001

Thoracotomy is associated with the recruitment of leukocytes into the ventricle within one week after the procedure.^25^ To test whether zCNTF injection elicits a similar response, we performed immunostaining against L-plastin, also called *lcp1*, which is a leukocyte-specific actin-bundling protein.^65,66^ Interestingly, the zCNTF-injected ventricles contained approx. twice more L-plastin-positive cells compared to control (Fig. 4a, e). This phenotype was reverted by treatment with Ruxolitinib, suggesting that the recruitment of immune cells to the injury site was dependent on JAK/STAT3 signaling. We concluded that a pulse delivery of exogenous zCNTF triggers responses that mimic preconditioning in the zebrafish ventricle.

Markers for specific leukocytes in the adult zebrafish are still subject to refinement.^67^ To distinguish between different types of leukocytes, we used the transgenic fish line, *mpeg1:EGFP*, which demarcates macrophages.^68^ To identify neutrophils, we performed immunofluorescence staining against Myeloperoxidase (Mpx, also abbreviated as Mpo) and Lysozyme (Lyz), both of which have been validated by several assays as myeloid-specific markers.^69^ We found that thoracotomy and zCNTF injection increased the number of *mpeg1:EGFP-*expressing cells by approx. 25- and 9-fold, respectively, compared to their controls (Fig. 4b, f). Furthermore, the number of Mpx- or Lyz-positive cells that co-expressed L-plastin displayed a 3-fold increase after thoracotomy and zCNTF injection compared to their controls (Fig. 4c, d, f). Taken together, these results indicate that both macrophages and neutrophils are recruited into the ventricle upon thoracotomy or intrathoracic CNTF injection.

### Injected CNTF mimics the preconditioning phenotype in regenerating zebrafish heart

**Fig. 5:**
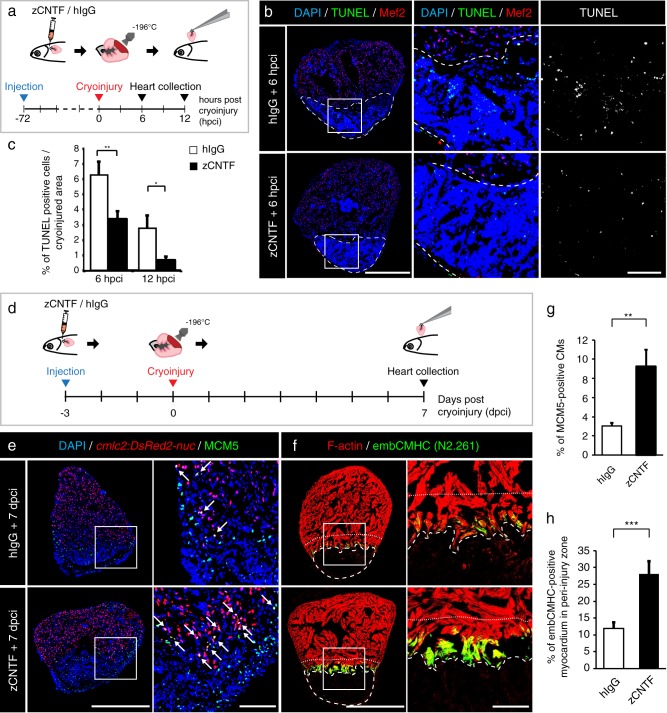
Administration of zCNTF reduces apoptosis and boosts initiation of heart regeneration after cryoinjury. **a** Experimental design for the apoptosis assay. zCNTF was injected into the pericardial cavity 72 h before cryoinjury. Hearts were collected at 6 and 12 h post-cryoinjury (hpci). **b** Transversal heart sections at 6 hpci stained for apoptotic cells using the TUNEL assay (green) and a myocyte nuclear marker Mef2 (red). Cryoinjured zone is encircled with a dashed line. Scale bar for the whole section, 500 μm; for the magnified area, 100 μm. **c** Quantification of TUNEL-positive nuclei at 6 and 12 hpci, in hearts injected with control proteins and zCNTF. *n* ≥ 4 hearts; ≥ 2 sections per heart; *P < 0.05; **P < 0.01. **d** Experimental design for assessment of the initiation of regeneration. zCNTF was injected into the pericardial cavity at 3 days before cryoinjury (dpci). Hearts were collected at 7 dpci. **e**, **f** Immunofluorescence staining of heart sections. Scale bar for the whole section, 500 μm; for the magnified area, 100 μm. **e** Transversal heart sections of transgenic fish *cmlc2:DsRed2-nuc* immunstained for MCM5 (green) display an enhanced number of proliferating CMs (arrows) after zCNTF injection. **f** Transversal heart sections of zCNTF/hIgG injected wild type fish immunostained for embCMHC (N2.261, green) and F-actin (red) comprise more abundant expression of embCMHC in the peri-injured ventricle within a distance of 100 μm from the wound tissue, which is delineated with a dotty line. **g** Quantification of MCM5-positive cardiac nuclei in hearts injected with control proteins and zCNTF. *n* ≥ 4 hearts; ≥ 2 sections per heart; *P < 0.05; **P < 0.01. **h** Proportion of the embCMHC-positive myocardium within the 100 μm peri-injury zone in hearts injected with control proteins and zCNTF. *n* ≥ 4 hearts; ≥ 2 sections per heart; ***P < 0.001

Preconditioning improved initiation of regeneration after ventricular cryoinjury, by rendering the heart more resilient to injury and by boosting CM proliferation.^29^ To determine if CNTF can reproduce these beneficial effects and act as a preconditioning stimulus, we performed injections of this protein prior to cryoinjury. In this model, the process of freezing and thawing destroys the cellular integrity, whereby fully disrupted cells rapidly die, whereas partially damaged cells may enter the apoptotic program within 24 h after the procedure.^30^ To assess the level of cell survival during this critical recovery period, we injected zCNTF into the pericardium 3 days before inducing ventricular damage, and collected hearts at 6 and 12 h post-cryoinjury (hpci) (Fig. 5a). A TUNEL assay of the ventricles revealed that apoptosis was reduced by 2- and 4-fold at 6 and 12 hpci, respectively (Fig. 5b, c). This finding suggests that exogenous zCNTF might improve cell survival upon partial damage.

To assess the effects of zCNTF on reactivation of the regeneration programs, we used *cmlc2:DsRed-nuc* transgenic fish and analyzed CM proliferation and dedifferentiation at 7 dpci (Fig. 5d). The assessment of MCM5 cell-cycle marker revealed a 3-fold increase in CM proliferation in regenerating hearts after zCNTF injection, compared to after hIgG injection (Fig. 5e, g). We have previously demonstrated that CMs of the peri-injury zone within 100 μm from the wound margin reactivate expression of embryonic cardiac myosin isoform (embCMHC, monoclonal antibody N2.261).^12,70^ Remarkably, the zCNTF-injected group contained a twice-larger embCMHC-positive area in the peri-injured myocardium compared to control, suggesting a more efficient CM dedifferentiation (Fig. 5f, h). Taken together, our results indicate that exogenous zCNTF increases cell survival after damage and enhances the entry into the regenerative program.

### *cntf* mutations abrogated the CM proliferation response following thoracotomy

**Fig. 6:**
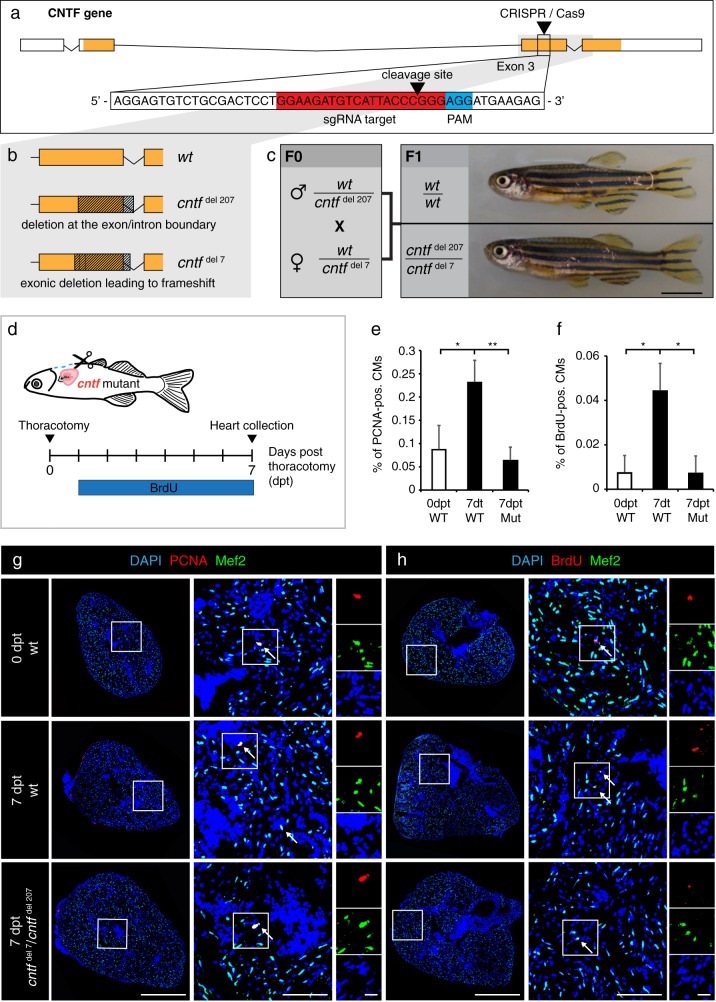
*Cntf* mutants fail to enhance CM proliferation after thoracotomy. **a** Schematic drawing of the *cntf* locus containing 4 exons. UTR (white); translated sequences (orange boxes). The sequence of 154-174 nucleotides (in red) flanking PAM sequence (in blue) in the 3^rd^ exon was targeted by the CRISPR/Cas9-sgRNA RNP complex. **b** Schematic drawing of the two deletions induced by CRISPR/Cas9. *cntf*^*del207*^ contains a deletion of 207 bp in the 3rd exon spanning the exon/intron boundary. *cntf*^*del7*^ comprises a deletion of 7 bp in the middle of the 3rd exon leading to a frameshift and a premature stop codon. **c** Genotypes and images of wild type and CNTF mutant siblings. F0 mosaic heterozygous candidates were crossed to obtain F1 trans-heterozygous mutants, which are viable without visible phenotype. Scale bar, 5 mm. **d** Experimental design. **e** Quantification of PCNA-positive cells among Mef2/DAPI-positive CM nuclei. *n* ≥ 3 hearts; ≥ 3 sections per heart; *P < 0.05, **P < 0.01. **f** Quantification of BrdU-positive cells among Mef2/DAPI-positive CM nuclei. *n* ≥ 3 hearts; ≥ 3 sections per heart; *P < 0.05. **g**, **h** Transversal heart sections of wt and *cntf* mutant fish at 7 dpt, immunostained against Mef2 (green, a myocyte nuclear marker) and cell proliferation markers, PCNA (**g**, red) or BrdU (**h**, red). All nuclei are labeled with DAPI (blue). Arrows indicate some proliferating CMs. Scale bar for the whole section, 500 μm; for the magnified area, 100 μm; for the zoom of magnified area, 20 μm

To genetically investigate the requirement of CNTF for thoracotomy-induced preconditioning, we generated mutant fish using CRISPR-Cas9. We injected wildtype embryos with Cas9-sgRNA ribonucleoprotein complexes (RNPs) that induce double strand breaks (DSB) in *cntf* between the 154^th^ and 155^th^ nucleotide of exon 3 (Fig. 6a). Two mutants were identified, namely *cntf*^*del207*^ with a 207 bp deletion that includes the exon/intron boundary, and *cntf*^*del7*^ with a 7 bp deletion that causes a frameshift, followed by a premature stop codon (Fig. 6b). The *cntf*^*del207*^ and *cntf*^*del7*^ mutations lead to alteration of the protein sequence after the 85^th^ and 88^th^ amino acid, respectively. After incrossing of these F0 crispants, we raised *cntf*^*del207*^/ *cntf*^*del7*^ trans-heterozygous zebrafish and their wild type siblings, which were identified by genotyping (Fig. 6c). We did not observe any phenotype in F1 adult mutant fish (Fig. 6c).

In order to assess preconditioning in the *cntf* mutant fish, we analyzed CM proliferation, immune cell recruitment and ColXII deposition in the hearts at 7 dpt. Compared to wildtype siblings, no difference was observed in the presence of L-plastin- and Mpx-positive cells, as well as in the infiltration of ColXII fibers in the ventricle (data not shown). To determine CM proliferation after thoracotomy, we used PCNA and BrdU-incorporation assays. Colocalization between PCNA and a myocyte marker Mef2 in DAPI-positive nuclei revealed approx. 3.5-times fewer proliferating CM in *cntf*^*del207*^/*cntf*^*del7*^ fish compared to their control siblings (Fig. 6e, g). Similarly, the mutant zebrafish displayed approx. 6-times less BrdU-labeled myocytes than control (Fig. 6f, h). These findings suggest that CNTF is required for stimulation of CM proliferation after thoracotomy-induced preconditioning.

## Discussion

The adult mammalian heart exhibits only very limited cardiomyocyte renewal, which is insufficient for regeneration of myocardial damage. It is therefore important to develop approaches to prevent the detrimental consequences of injury. After infarction, reperfusion is mandatory to salvage the ischemic myocardium. Paradoxically, cardiomyocyte death occurs not only during ischemia but also during ‘myocardial reperfusion injury’. There is currently no cardioprotection stronger than that elicited by the preconditioning phenomena.^32^ Preconditioning is an intriguing phenomenon, whereby endogenous cellular survival and pro-regenerative programs are induced by transient exposure to noxious stimuli.^31,36^ Consequently, activated protective mechanisms increase the resilience of tissues to further harmful injuries. Preconditioning is a powerful intervention known for reducing infarct size and improving clinical outcomes in patients with ischemic heart disease.^35,71^ Despite its potential in regenerative medicine, the cascade of events invoking protective programs still remains unclear. In this study, we used transcriptional profiling of the zebrafish ventricle to determine differentially expressed genes after cardiac preconditioning, whereby chest incision activates pro-regenerative programs in the heart even in the absence of myocardial damage.^29^ As in mammalian preconditioning, we identified upregulation of genes involved in cytoprotection, epithelial-to-mesenchymal transition and extracellular matrix remodeling. This finding indicates that the molecular players of preconditioning might be conserved among vertebrates.

Our analysis revealed expression changes of several epicardium-derived signaling factors, which have previously been identified in epicardial cell transcriptome sequencing in zebrafish.^72^ Specifically, we found that thoracotomy resulted in downregulation of *neuregulin 2a* (*nrg2a*), whose homolog *nrg1* was reported in *tcf21* *+* perivascular cells.^73^ Several epicardial signaling factors were upregulated in preconditioned hearts, including a CXC-motif chemokine ligand *cxcl12*,^22^ *insulin growth factor 2b* (*igf2b*),^23,74^ *thymosin beta* and *midkine A*.^75^ These signaling molecules might regulate CM proliferation, survival and leukocyte recruitment. Here, we focused our analysis on the epicardium activation and the LIFR/GP130 signaling pathway during preconditioning.

We identified a few mediators of epithelial-to-mesenchymal transition (EMT), such as regulators of the plasma membrane organization annexins (*anxa1a, anxa2a*)^76,77^ and a master transcription factor *slug* (also called *snai2*).^78^ *In-situ* hybridization of several candidate genes and immunofluorescence analysis of *ET27:EGFP* transgenic-reporter fish revealed that the epicardium is stimulated after thoracotomy. We found that the thoracotomy-activated epicardial cells invade the underlying myocardium that is associated with ColXII deposition. This type of collagen is particular because it does not form stereotypic fibers on its own, but regulates the size, spacing and interconnection between ECM fibrils.^53^ Indeed, ColXII may modulate the matrix arrangement and its morphogenetic flexibility, especially under biophysical stress during tissue restoration and homeostasis.^54,56,79,80^ The requirement of ColXII in the zebrafish heart needs further investigation.

The pathway analysis of the differentially expressed genes revealed that multiple members of the LIFR/GP130 receptor pathway were enriched at 1 dpt. In mammalian models, downstream JAK1/STAT3 signal transducers are activated upon cardiac stress, such as pressure overload, hypoxia or injury.^81^ Furthermore, STAT3 has been shown as a mediator of cardioprotective signaling in the pig and mouse heart.^82,83^ In the zebrafish heart, overexpression of dominant negative STAT3 has been shown to restrict CM proliferation during regeneration, but not during normal growth.^84^ Our study revealed that *cntf*, a cytokine of LIFR/GP130 signaling,^40^ is transcriptionally induced after thoracotomy. Although our *in-situ* hybridization analysis indicated that the epicardium expresses *cntf*, other tissues could also contribute to production of extracellular CNTF, which might be distributed in a paracrine or systemic manner.

We showed that a single injection of zebrafish CNTF into the uninjured pericardial cavity was sufficient to mimic various preconditioning responses, including stimulation of the cell cycle entry of CMs and non-CMs, expansion of ColXII fibers into the myocardium and recruitment of macrophages. The number of dsRed/MCM5-double positive cells was lower in our control fish than in our previous study.^70^ This discrepancy in the proliferation dynamics might be caused by epigenetic changes in the *cmlc2:DsRed-nuc* strain. However, as we used sibling fish for hIgG and CNTF injections, the comparison between both groups should reveal the direct effects of the injected molecules. Importantly, exogenous CNTF exerted beneficial effects in the cryoinjury model. Pre-injected fish displayed reduced apoptosis as assessed at 6 and 12 hpci, more proliferation of CMs at 7 dpci, and a higher expression of the embryonic isoform of the myosin heavy chain. Thus, similarly to thoracotomy, one injection of CNTF is sufficient to reproduce preconditioning in the zebrafish heart.

Since CNTF possesses a wide spectrum of biological actions,^40,42,43^ it remains unclear whether its beneficial effects observed in the heart are direct or indirect. In this study, we were not able to visualize STAT3 phosphorylation by immunofluorescence, which is essential to monitor the signal-activated cells. The inhibition of JAK/STAT3 in the zebrafish heart was sufficient to suppress the effects of CNTF, but we cannot exclude that other downstream signaling cascades contribute to CNTF-mediated preconditioning. To determine whether the endogenous *cntf* gene is required for the preconditioning effects of thoracotomy, we generated mutant zebrafish using the Crispr/Cas9 method. Two alleles contained deletions that disrupt the protein sequence after the 85^th^ and 88^th^ amino acid. The missing part of the protein is predicted to comprise one out of the four α-helixes and 5 protein binding sites.^85^ Thus, it is likely that the mutated alleles produce truncated non-functional proteins. The trans-heterozygous mutant fish did not display any developmental defects. Nevertheless, adult mutant fish failed to enhance CM proliferation upon thoracotomy. Thus, *cntf* seems to be essential for stimulation of the pro-regenerative program after preconditioning. Further studies could be conducted in the future to address the role of *cntf* during zebrafish heart regeneration after cryoinjury.

Mouse knockout and human genetic studies have revealed that the *cntf* gene is not essential during development or for healthy life,^86,87^ but the CNTF protein can exert protective effects for neural tissues when exogenously provided.^62^ Ectopically administrated CNTF can decrease progression of motor neuron diseases in rodent and primate models.^88,89^ CNTF can act as a neuroprotective or pro-regenerative agent in retinas of mice and zebrafish.^63,90^ This cytokine also plays a metabolic role in hepatocytes, in which it stimulates lipid metabolism,^91^ and adipocytes, where it increases insulin sensitivity, decreases fatty acid synthesis and stimulates production of anti-obesogenic leptin.^92^ In cultured skeletal muscle cells, CNTF promotes myoblasts proliferation and inhibits myogenic differentiation.^93,94^ To our knowledge, the role of this exogenous cytokine has not yet been reported in the vertebrate heart. Thus, our findings on the cardioprotective and pro-regenerative effects of CNTF in the zebrafish heart allow new insights into the function of this cytokine in vertebrates. The mechanisms of the protective tissue response are probably evolutionary conserved. The zebrafish heart model will provide a deeper comprehension of the cardioprotective mediators that is necessary to guide pharmacological research that aims to mimic preconditioning processes.

## Methods

### Zebrafish lines and animal use

Wild type AB (Oregon) and transgenic adult zebrafish aged 6 to 18 months were used in this study. Genetically modified lines were: *Tg(cmlc2:DsRed2-Nuc)*,^59^ *Tg(mpeg1:EGFP)*,^68^ *Tg(ET27:EGFP)*.^55^ The trans-heterozygous *cntf*^del207^/ *cntf*^*del7*^ fish were at the age of 3 months old. All assays were performed using different animals that were randomly assigned to experimental groups. The exact sample size (n) was described for each experiment in the figure legends, and was chosen to ensure the reproducibility of the results. During invasive procedures and imaging, fish were anaesthetized with buffered solution of 0.6 mM tricaine (MS-222 ethyl-m-aminobenzoate, Sigma-Aldrich) in system water. Animal procedures were approved by the cantonal veterinary office of Fribourg, Switzerland.

### Animal procedures

For all interventions, anesthetized fish were placed ventral side up in a damp sponge under the stereomicroscope.

Thoracotomy were performed by cutting a 1-2 mm incision through the chest with iridectomy scissors (Roboz Surgical Instrument Co.) The beating heart was well visible, and no extensive bleeding occurred during the thoracotomy. It was not necessary to suture the wound, as the healing process occurs spontaneously within a week.^29^

Ventricular cryoinjuries were performed according to our video protocol.^95^ Briefly, the ventricular wall was directly frozen by applying for 23-25 sec a stainless steel cryoprobe (custom-made) precooled in liquid nitrogen. To stop the freezing of the heart, system water at room temperature was dropped on the tip of the cryoprobe, and fish were immediately returned into water.

Intrathoracic microinjections were performed using pulled glass needles TW100F-6 (World Precision Instruments) and an Eppendorf Femto Jet microinjector. To ensure the reproducibility of the injections and monitor spreading of the liquid under the chest of the fish, 10% Phenol Red was added to the solution. Injections of 2.5 μl solution into the pericardial cavity were guided by observation under the stereomicroscope (Suppl. Fig. S6). Injections were performed with caution to avoid any direct contact between the needle and the heart. If the heart was touched or punctured by the needle, the fish were excluded from experiments.

For the bromodeoxyuridine (BrdU) incorporation assay, the animals were maintained in 5 mg/ml BrdU (B5002; Sigma-Aldrich) for 6 days at 22 °C starting from 1 dpt.

To collect the heart for fixation, fish were euthanized in 0.6 mM tricaine solution. An incision was made above the heart through the branchial cartilage and the heart was pulled from the body cavity as shown in the video protocol.^95^ For analysis of leucocytes, the hearts were prefixed for 15 min prior to collection by injecting 5 μl of 2% paraformaldehyde into the pericardial cavity of euthanized animals. This prefixation was performed to avoid corruption of the results by immune cells that are carried in the blood and that adhere to the surface of the ventricle at the moment of heart collection.

### Drug treatments

The JAK1/2 kinases inhibitor Ruxolitinib (Selleckchem) was dissolved in DMSO at a stock concentration of 1 mM and used at a final concentration of 1 μM. The TGFβ type I receptor inhibitor SB431542 (Tocris) was dissolved in DMSO at a stock concentration of 20 mM and used at a final concentration of 20 μM. The FGFR1 inhibitor PD173074 (Tocris) was dissolved in DMSO at a stock concentration of 10 mM and used at a final concentration of 10 μM. Control animals were kept in water with 0.1% DMSO. Zebrafish were treated with drugs at a density of 3 fish per 100 ml of water. During the treatments, fish were fed and solutions were changed every second or third day.

### CRISPR-Cas9-induced *cntf* mutation

Mutant fish were generated as previously described.^96^ The targeting sequence was: GGAAGATGTCATTACCCGGGAGG (PAM underlined). Two *cntf* alleles were established. First, *cntf*^*del207*^ contains a deletion of 207 bp, corresponding the sequence between 144 and 351 bp of *cntf* gene counted from the beginning of the 3^rd^ exon (NM_001145632). This deletion covers a large part of the coding exon and the exon/intron boundary, and is predicted to affect the downstream sequence. Second, *cntf*^*del7*^ lacks 7 bp between 151 and 158 bp of *cntf* gene counted from the beginning of the 3^rd^ exon. This deletion results in a frameshift followed by a premature stop codon. *cntf*^*del207*^ / *cntf*^*del7*^ trans-heterozygous fish at the age of 3 months were used for this study.

### Reporting summary

Further information on experimental design is available in the Nature Research Reporting Summary linked to this article.

## Supplementary information

Supplementary material

Suppl. Data S1

Suppl. Data S2

Suppl. Data S3

Reporting Summary

## Data Availability

The authors declare that all data supporting the findings of this study are available within the article and its Supplemental Material files, or from the corresponding author upon reasonable request.

## Supplementary information

**Supplementary information** accompanies the paper on the *npj Regenerative Medicine* website (10.1038/s41536-019-0064-9).
