# Supplementary material for: Ciliary neurotrophic factor stimulates cardioprotection and the proliferative activity in the adult zebrafish heart
Source: NPJ Regen Med. 2019 Jan 24;4:2. doi: 10.1038/s41536-019-0064-9 (PMC6345746; doi:10.1038/s41536-019-0064-9)
Supplement: Supplementary file 1 — Supplementary material [file 41536_2019_64_MOESM1_ESM.pdf]

SUPPLEMENTAL MATERIAL

Supplemental Methods

Immunofluorescence analysis

Adult hearts were fixed in 2% paraformaldehyde overnight at 4°C, followed by washes in PBS (3 × 10 min each). Heart specimens were equilibrated in 30% sucrose at 4°C, embedded in tissue freezing media (Tissue-Tek O.C.T.; Sakura) and cryosectioned at a thickness of 16 µm. Sections were collected on Superfrost Plus slides (Fisher) and stored in tight boxes at −20 °C. Before use, slides were brought to room temperature for 10 min and the area with sections was encircled with PAP Pen (Vector). Then, the slides were transferred to coplin jars containing 0.3% Triton-X in PBS (PBST) for 10 min at RT. Antibody staining was performed in a humid chamber. Blocking solution (5% goat serum in PBST) was applied on the sections for 1 h at RT. Subsequently, sections were covered with approx. 200 µL of primary antibody diluted in blocking solution and incubated overnight at 4 °C. On the next day, they were washed in PBST in coplin jars for 1 h at RT and again transferred to the humid chamber for incubation with secondary antibodies in blocking solution. The slides were washed in PBST for 1 h at RT and mounted in 90% glycerol in 20 mM Tris pH 8 with 0.5% N-propyl gallate.

The following primary antibodies were used: chicken anti-L-Plastin at 1:1000 (kindly provided by P. Martin, Bristol) [1], chicken anti-GFP at 1:2000 (GFP-1010, Aves Labs), mouse anti-embCMHC (N2.261) at 1:50 (developed by H.M. Blau, obtained from Developmental Studies Hybridoma Bank), mouse anti-tropomyosin at 1:100 (developed by J. Jung-Chin Lin and obtained from Developmental Studies Hybridoma Bank, CH1), mouse anti-phospho-Histon H3 (Ser10) clone 3H10 (pH3) (05-806; Upstate); rabbit anti-Mef2 at 1:50 (sc-313, Santa Cruz Biotech.), rabbit anti-MCM5 at 1:500 (kindly provided by Soojin Ryu, Heidelberg) [2], mouse anti-PCNA at 1:500 (Dako M0879), rat anti-BrdU at 1:200 (Abcam ab63261), rabbit anti-Mpx at 1:500 (GTX128379 GeneTex), rabbit anti-Lysozyme at 1:200 (GTX132379 GeneTex), guinea pig anti-ColXIIa (kindly provided by F. Ruggiero, Lyon) [3]. The secondary antibodies (at 1:500) were Alexa conjugated (Jackson ImmunoResearch Laboratories). Phalloidin-Atto-565 (94072, Sigma), Atto-647N (65906, Sigma) and 405 (CruzFluor™) were used at 1:500. DAPI (Sigma) was applied to label nuclei.

Image analysis and quantification

After antibody staining, cardiac tissue imaging was performed at 20x magnification with confocal microscopes (Leica TCS-SP5 and Leica TCS-SPE-II). n represents the number of fish used in the experiment. At least 3 different pictures were taken for each heart. Image analyses were performed

using ImageJ 1.49c software. To quantify the number of proliferating cells, the images of the nuclear cell-cycle marker (MCM5) were superimposed with the images of DAPI and *cmlc2:DsRed2-nuc*, which labels cardiac nuclei. The number of proliferating non-CMs was obtained by subtracting the number of MCM5/*cmlc2:DsRed2-nuc*-positive nuclei from the number of MCM5/DAPI-positive nuclei. For quantification of dedifferentiating CMs after cryoinjury, embCMHC positive area was superimposed with the F-actin-positive area within 100 µm from the wound margin. The number of apoptotic nuclei was assessed by the superimposition of DAPI with the TUNEL labeling. The L-Plastin-positive area was normalized to the total area of the ventricular section. For quantification of specific populations of leucocytes, L-plastin was superimposed with either *mpeg1:EGFP*, Mpx or Lyzozyme and normalized to the area of heart sections. Error bars correspond to standard error of the mean (SEM). Significance of differences was calculated using unpaired Student's t-test. Statistical analyses were performed with the Microsoft Excel software.

Terminal deoxynucleotidyl transferase dig-dUTP nick end-labeling (TUNEL)

For TUNEL reactions, the cryosections were postfixed for 10 min in 1% formalin, washed twice for 5 min in PBS and pretreated in precooled ethanol:acetic acid 2:1 for 5 min at -20°C. After washing in PBS, DNA breaks were elongated with Terminal Transferase (Roche) and Digoxigenin-dUTP solution (Roche). The reaction was stopped by incubation in 300 mM NaCl, 30 mM sodium citrate for 10 min, followed by washing in PBS. The staining with anti-digoxigenin fluorescein conjugated was performed according to manufacturer protocol (Roche).

***In-situ* hybridization**

The forward (F) and reverse (R) primers (5' to 3') used to synthesize the template for the ISH probes are listed (Supplemental Table S1). To synthesize the anti-sense probes, a sequence of T3 RNA polymerase promoter (attaaccctcactaaagggaga) was added to the 5' end of the reverse primers. The digoxigenin-RNA labeling mix (Roche) was used to generate the probe, which after purification was dissolved in hybridization solution and stored at -20 °C.

Table S1. Primers for in situ-hybridization and qRT-PCR analysis

Application	Gene	Gene ID	Forward primer 5'->3'	Reverse primer 5'->3'	Product length
qRT-PCR	*b-actin*	ENSDARG00000037870	TTGGCAATGAGAGGTTCAGG	TGGAGTTGAAGGTGGTCTCG	55 bp
	clcf	ENSDART00000109896	AGAGCTCCCTCCTGCACTTC	ACCGGTCCTCCATAGTAGCC	94 bp
	cntf	ENSDARG00000074662	CCACAAGGAATGCATCCAAC	TCCTTCCACTGGTGAAGACG	73 bp
	lif	ENSDART00000137503	GTTCGCCACATGGCTGTAAG	TTTCAGCTGGCTCAGGAATG	79 bp
in situ	*adh8a*	ENSDART00000008498	TGCAAGTTCTGCAAGTGTC	GATCATGGAATGCAAACAGC	918 bp
	anxa1a	ENSDART00000031665	AGGAACGGTGAAGCCCTATG	GCCACACAATGCTAGCAGG	934 bp
	anxa2a	ENSDART00000011967	GATGCTGCCAGGATTGAGAC	GGACCTCAGAGCTCCAGTTC	940 bp
	cntf	ENSDARG00000074662	ACAAACGGTCGTCATGGGT	TTCTTGTCCGTTGGGACACT	547 bp
	cystatin	ENSDARG00000074425	GATTCACTGTCGGGTTTGGG	ATTGGGTCCATGGTGACCTC	541 bp
	col12a1b	ENSDARG00000019601	AAGGCGGAAATACAATGACG	TCAAACGCATAGAGCTGTGG	945 bp
	mmp13a	ENSDART00000011573	TTCACTCGCATCTACAGCGG	CCTCTGTACTGGAAGGCTGC	875 bp
	mdka	ENSDARG00000036036	AGCCACTCCTCTGCTGTTTC	GTACCAGCTGAAGGGCAAAG	802 bp
	slug	ENSDART00000058571	TCAGCATGCCTCGTTCATTC	ACGTGTAGCATCTTGGTTGG	955 bp
	socs3b	ENSDART00000037904	AGTATCAGATGGTGCTCGCG	CGAGTGTCTCCCGATCGATG	921 bp
	thymB4	ENSDART00000131861	GCTGACTATAGCTCTCCAGCATC	TTAAACCCTGTCTCGCATCC	495 bp
	timp2b	ENSDART00000166855	AACTATCGCTCTCCGAAGTTTG	TGGGGTTCTTAAATGAAGGATG	771 bp
	txn	ENSDART00000064789	GCTCAAACGACACACGAGC	GTTTTCATTTCATACAAAGCCAACA	643 bp
	cxcl12a	ENSDART00000053946	AGTTCCTCCACACACCCAAC	AAACACGGAGCAAACAGGAC	452 bp

In-situ hybridization was performed on heart cryosections. Hearts were fixed overnight at 4°C in 4% paraformaldehyde, then washed twice in PBS, once in PBS/methanol and in methanol for 10 min each, and finally transferred to methanol. Fixed material was stored at -20 °C until processed for cryosectioning, which was performed as for immunofluorescence. Before use, slides were brought to room temperature for 10 min, and incubated in hybridization solution (50% formamide, 5 x SSC, 1 x Denhardt's solution, 10 % dextran sulphate, 0.1 mg/ml yeast tRNA; all from Sigma-Aldrich) for 1 h at RT and for 1 h at 60°C. Then, they were placed into tight boxes with wet paper towels, 200 µl of probe diluted in hybridization solution (1:200) was applied on each slide that was then covered with a plastic coverslip (ApopTag, Merck Millipore) and placed in an incubator at 60 °C overnight. The probes were used at 1.6 – 4.8 ng/µl final concentration. On the next day, slides were transferred to a coplin jar and soaked for 5 min with prewarmed 5 x SSC (Saline-sodium citrate) to allow the removal of cover slips, and washed twice with 5 x SSC for 30 min and once with 0.2 x SSC for 1 h, each step at 60°C. The final wash was done with 0.2 x SSC for 5 min at RT. Slides were allowed to dry for a few minutes at RT and sections was encircled with Pap Pen, and left for 15 min for drying. Slides were placed into humidified boxes, and 1x blocking reagent solution (Roche) in maleic acid buffer (100 mM Maleic Acid, 150 mM NaCl, 0.2% Tween-20, pH 7.5; all from Sigma-Aldrich) was applied on sections for 1 h at RT. Subsequently, 250 µl of anti-dig-alkaline phosphatase (AP) antibody (Roche; diluted 1:4000 in blocking reagent solution) was applied on sections for 2 h at RT. Slides were rinsed and washed twice for 30 min in maleic acid buffer in coplin jars. Then, slides were washed twice in AP-buffer (100 mM Tris HCL pH 9.5, 100 mM NaCl, 50 mM MgCl2, and 0.2% Tween 20) for 5 min. To prepare the staining reagents, NBT and BCIP compounds (Roche) were diluted in AP-buffer according to manufacturer's instruction

with addition of 10 % Polyvinyl alcohol (Sigma-Aldrich). The staining reaction was performed in a humidified box at 37 °C. The reaction was monitored under the microscope, and was stopped by washes in coplin jars with PBS for 15 min, 70 % ethanol for 1 h, and 15 min in PBS. The slides with fins were mounted in Aquatex mounting medium (Merck Millipore).

Quantitative real time PCR

RNA was extracted according to the Trizol reagent manual (Life Technologies) with the use of MaXtract High Density tubes. cDNA was synthetized with the Super-Script-II Reverse-Transcriptase (Invitrogen) using 1.5 µg of RNA. The primers used for different amplifications are listed in Supplementary Table S1.

Expression and purification of zebrafish CNTF

The codon-optimized (for expression in *E. coli*) zCNTF gene (NM_001145632.1) was generated by DNA synthesis (Eurofins) and subcloned into pGEX-4-3T (GE Healthcare) for expression as an N-terminally tagged glutathione S-transferase (GST) fusion protein in BL21(DE3) (Novagen) *E. coli* cells. Cells were grown in 400 ml of lysogeny broth (LB) medium at 37°C and protein expression was induced at an optical density (OD_{600}) of around 0.6 by the addition of IPTG to a final concentration of 0.5 mM. After 6 h of growth at 18°C (OD_{600} above 2), cells were harvested and stored at -80°C. Then, cells were resuspended in 25 ml ice-cold lysis buffer (50 mM Tris-HCl pH 7.5, 200 mM NaCl, 1.5 mM MgCl2, 5% glycerol) and lysed using a M-110L Microfluidizer (Microfluidics). The lysate was adjusted by the addition of 300 µl 10% NP-40 to 0.1% NP-40. An aliquot of 50 µl of total extract (sample T) was taken and mixed with 50 µl of 6x loading buffer, whereas the remaining lysate was centrifuged at 4°C for 20 min at 14'000 rpm. The supernatant (soluble extract) was collected and an aliquot of 50 µl was mixed with 50 µl of 6x loading buffer (sample S). The insoluble pellet was resuspended in 3 ml of lysis buffer containing 0.1% NP-40 and 5 µl thereof were mixed with 45 µl of lysis buffer and 50 µl of 6x loading buffer (sample P). GST-zCNTF was purified from the soluble extract by incubation with 300 µl Glutathione (GSH) Superflow beads (Qiagen) for 3h at 4°C on a rotating wheel. The beads were harvested by centrifugation for 1 min at 1800 rpm, transferred to an 1.5 ml Eppendorf tube, and subsequently washed five times with 1 ml of lysis buffer containing 0.1% NP-40. After the last centrifugation, the supernatant was removed and 1 ml of lysis buffer was added to the beads; then, 5 µl of this dilute bead slurry were added to 45 µl of lysis buffer and 50 µl of 6x loading buffer. To elute zCNTF from the GSH agarose beads by thrombin cleavage, beads were incubated overnight at RT in PBS pH 7.3 containing 20 units of thrombin (Sigma) and the reaction was stopped with 1 mM PMSF. The beads were collected by centrifugation and the supernatant (zCNTF eluate) was transferred to a fresh

Eppendorf tube. The pelleted beads were resuspended in 1 ml lysis buffer and 5 µl thereof was added to 45 µl of lysis buffer and 50 µl of 6x loading buffer. A 5µl aliquot of the zCNTF eluate (sample E) was mixed with 45 µl of lysis buffer and 50 µl of 6x loading buffer. Protein samples (5 µl of samples T, P, S, and E) were separated on a NuPAGE 4-12% Bis-Tris 15-well gel (Novex), run in 1x MES SDS running buffer at 150 V for 90 min, and subsequently stained with Brilliant Blue G Coomassie (Sigma).

The identity of the proteins contained in the indicated bands was determined by mass spectrometric analysis of peptides obtained by digestion with trypsin as previously described [4].

CRISPR-Cas9-induced *cntf* mutants

The CNTF sgRNAs were designed using CHOPCHOP (http://chopchop.cbu.uib.no/index.php) [5,6]. This sequence was selected because of its convenience for further screening by the XmaI (NEB) restriction enzyme, which is specific for the CCCGGG sequence.

sgRNA was synthetized using oligo-based template generation and *in vitro* transcription as previously described (ref) using the following primers:

GAAATTAATACGACTCACTATA-N20-NGG GTTTTAGAGCTAGAAATAGC, where N20 indicates the sgRNA target sequence, and the invariant sgRNArev:

AAAAGCACCGACTCGGTGCCACTTTTTCAAGTTGATAACGGACTAGCCTTATTTTAACT TGCTATTTCTAGCTCTAAAAC-3′ (PAGE-purified, Microsynth). Primer extension was performed using Phusion polymerase (NEB), followed by column purification and *in vitro* transcription using SP6 RNA polymerase (NEB). sgRNAs were assembled with EnGen Cas9 NLS, S. pyogene (NEB) into ribonucleopeotein complexes (RNP) solubilized with 300 mM KCL as described [7] and were injected into 1-cell stage embryos of the AB wildtype strain. Injected animals were screened by amplifying a 1166 bp fragment of the locus using PCR primers:

cntf Fw : TTCACCAGTGGAAGGAGGTC

cntf Rv : CTTAGTTTCCCAGCACCTCTT

As the target site comprises XmaI restriction enzyme site, digestion of the amplified fragment was performed to prescreen the animals with disrupted sequence. Indeed, in case of double strands break (DSB)-induced mutation, the site CCCGGG is likely not being recognized by the restriction enzyme (XmaI). Two *cntf* alleles were found, as described in the main manuscript, and used for raising F1 progeny. Adult sibling fish were genotyped by sequencing to identify $cntf^{del207}$ / $cntf^{del7}$ trans-heterozygous and wild type animals.

High throughput sequencing and data analysis

Each experimental condition (0 dpt, 1 dpt and 7 dpt) was carried out in triplicate. For each experimental sample, ten fish were used for the RNA extraction. Hearts were homogenized with Tissue Lyser LT, the samples were transferred in MaXtract High Density tubes (Qiagen) before the addition of GenElute-LPA (Sigma), a synthetic polymer, which acts as a carrier for DNA/RNA. RNA was then isolated according to the Trizol reagent manual (Life Technologies) and purified according to the RNeasy MinElute Cleanup Kit manual (Qiagen). The quality of the RNA samples was tested using an Agilent 210 Bioanalyzer. RNA libraries were prepared according to the TruSeq RNA Sample Preparation v2 Guide (Illumina) and single-ends RNA sequencing was performed with an Illumina HiSeq2500 machine. Each RNAseq sample yielded between 2.4 mio and 3.5 mio reads of 100 bp in length. Reads were first checked for sequence quality (FastQC, www.bioinformatics.babraham.ac.uk/projects/fastqc). The last 20 bp of the reads were trimmed due to bad quality. The reads were then mapped to the zebrafish (*Danio rerio*) reference genome (version 9) using the spliced mapping approach implemented in TopHat2 [8]. Between 82-86% of reads were mapped to the *Danio rerio* genome. For each gene (annotation version 9.75) the number of reads mapping to it were counted using the program HTSeq-count (www.w-huber.embl.de/users/anders/HTSeq version 0.5.4p3). To test for significant differences of the expression levels between treatments the R-package DESeq was used [9].

The zebrafish gene identifiers of the differentially expressed genes (adj-p-value <0.05) were first converted to orthologous human identifiers using a custom made Perl script. The orthologs list was obtained from the HCOP database (http://www.genenames.org/cgi-bin/hcop) of the HUGO Gene Nomenclature Committee [10]. Some missing orthologs were rescued manually by searching in the BioMart database of ENSEMBL [11]. The humanized gene identifiers list and their corresponding fold changes were imported into the web-based integrative software GeneGo Metacore suite (v 6.18 build 65505; Thomson Reuters, St. Joseph, MI) for network analysis. MetaCore was used for pathway enrichment, network enrichment and GO terms enrichment analysis using the differentially expressed genes (enrichment p-value <0.05). The heat maps were generated with an R script using ggplot package after log transformation of the fold changes.

Supplemental References

1 Redd, M. J., Kelly, G., Dunn, G., Way, M. & Martin, P. Imaging macrophage chemotaxis in vivo: Studies of microtubule function in zebrafish wound inflammation. *Cell Motility and the Cytoskeleton* **63**, 415-422, doi:10.1002/cm.20133 (2006).

2 Chapouton, P. *et al.* her5 expression reveals a pool of neural stem cells in the adult zebrafish midbrain. *Development* **133**, 4293-4303, doi:10.1242/dev.02573 (2006).

3 Bader, H. L. *et al.* Zebrafish collagen XII is present in embryonic connective tissue sheaths (fascia) and basement membranes. *Matrix Biol* **28**, 32-43, doi:10.1016/j.matbio.2008.09.580 (2009).

4 Diedrich, B. *et al.* Discrete cytosolic macromolecular BRAF complexes exhibit distinct activities and composition. *The EMBO Journal* **36**, 646-663, doi:10.15252/embj.201694732 (2017).

5 Labun, K., Montague, T. G., Gagnon, J. A., Thyme, S. B. & Valen, E. CHOPCHOP v2: a web tool for the next generation of CRISPR genome engineering. *Nucleic Acids Research* **44**, W272-W276, doi:10.1093/nar/gkw398 (2016).

6 Montague, T. G., Cruz, J. M., Gagnon, J. A., Church, G. M. & Valen, E. CHOPCHOP: a CRISPR/Cas9 and TALEN web tool for genome editing. *Nucleic Acids Research* **42**, W401-W407, doi:10.1093/nar/gku410 (2014).

7 Burger, A. *et al.* Maximizing mutagenesis with solubilized CRISPR-Cas9 ribonucleoprotein complexes. *Development* **143**, 2025-2037, doi:10.1242/dev.134809 (2016).

8 Kim, D. *et al.* TopHat2: accurate alignment of transcriptomes in the presence of insertions, deletions and gene fusions. *Genome Biology* **14**, R36, doi:10.1186/gb-2013-14-4-r36 (2013).

9 Anders, S. & Huber, W. Differential expression analysis for sequence count data. *Genome Biology* **11**, R106, doi:10.1186/gb-2010-11-10-r106 (2010).

10 Gray, K. A., Yates, B., Seal, R. L., Wright, M. W. & Bruford, E. A. Genenames.org: the HGNC resources in 2015. *Nucleic Acids Research* **43**, D1079-D1085, doi:10.1093/nar/gku1071 (2015).

11 Kinsella, R. J. *et al.* Ensembl BioMarts: a hub for data retrieval across taxonomic space. *Database* **2011**, bar030-bar030, doi:10.1093/database/bar030 (2011).

Supplemental Data

Supplemental Data 1. Differentially expressed genes at 1 and 7 dpt. Up- and downregulated genes are listed on separate excel sheets for comparison between 0 dpt versus 1 dpt and 0 dpt versus 7 dpt. Each gene is annotated with the Gene Ontology (GO) term. (Excel sheets)

Supplemental Data 2. Common differentially expressed genes at 1 and 7 dpt.

Two excel sheets show the expression change between 0 dpt versus 1 dpt and 0 dpt versus 7 dpt for the common genes. Each gene is annotated with the Gene Ontology (GO) term. (Excel sheets)

Supplemental Data 3. The LIFR/GP130 signalling components are transcriptionally upregulated after preconditioning.
(Excel sheets)

SUPPLEMENTAL FIGURES

Supplemental Fig. S1

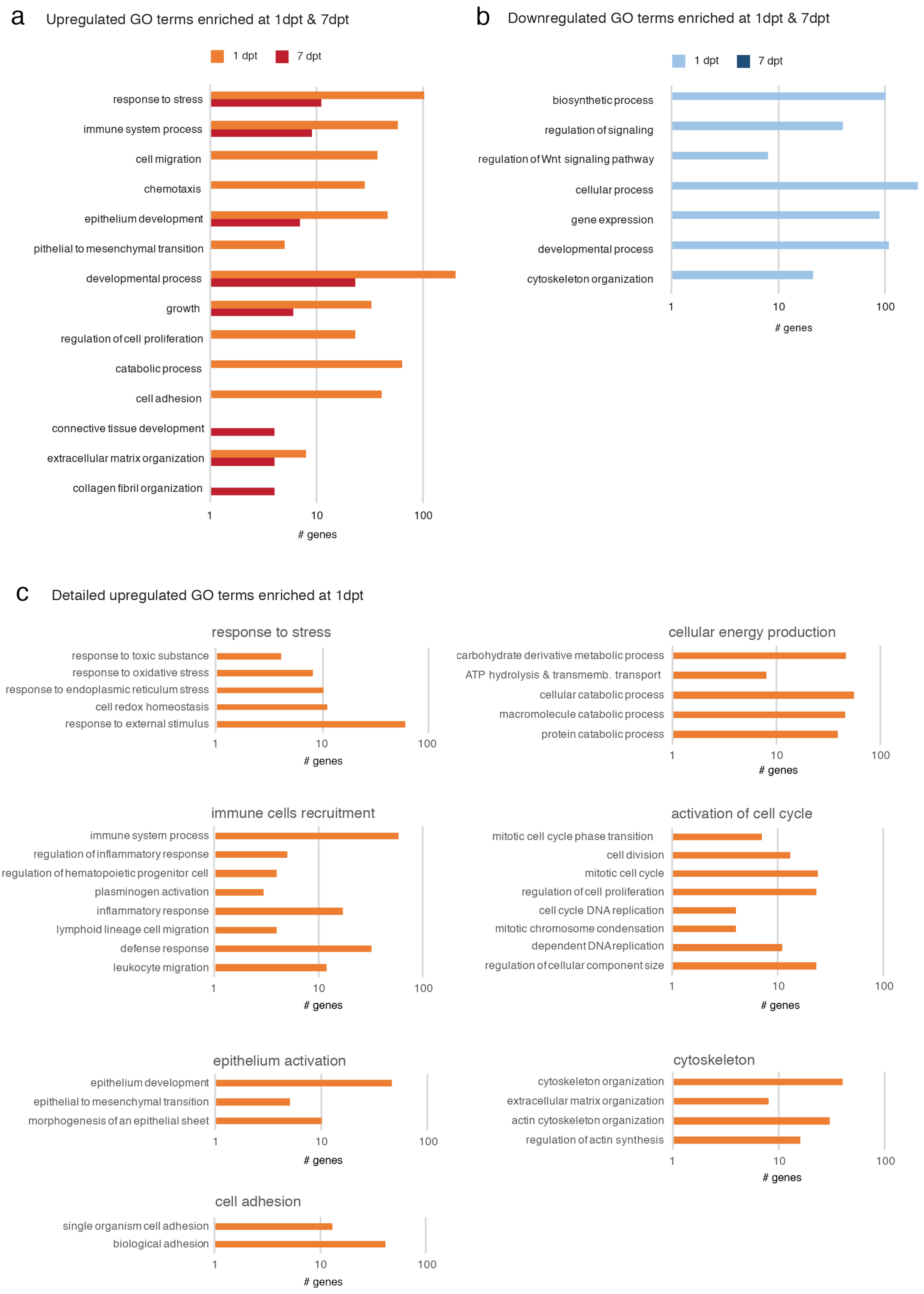

Fig. S1. Gene Ontology (GO) enrichment analysis of differentially expressed genes at 1 and 7 dpt.
(a-c) Differentially expressed genes (p-value ≤ 0.01 and $\log2 \geq 1$ (upregulated genes) or $\log2 \leq -1$ (downregulated genes) were categorized by clusters based on GO terms (biological processes).

Supplemental Fig. S2

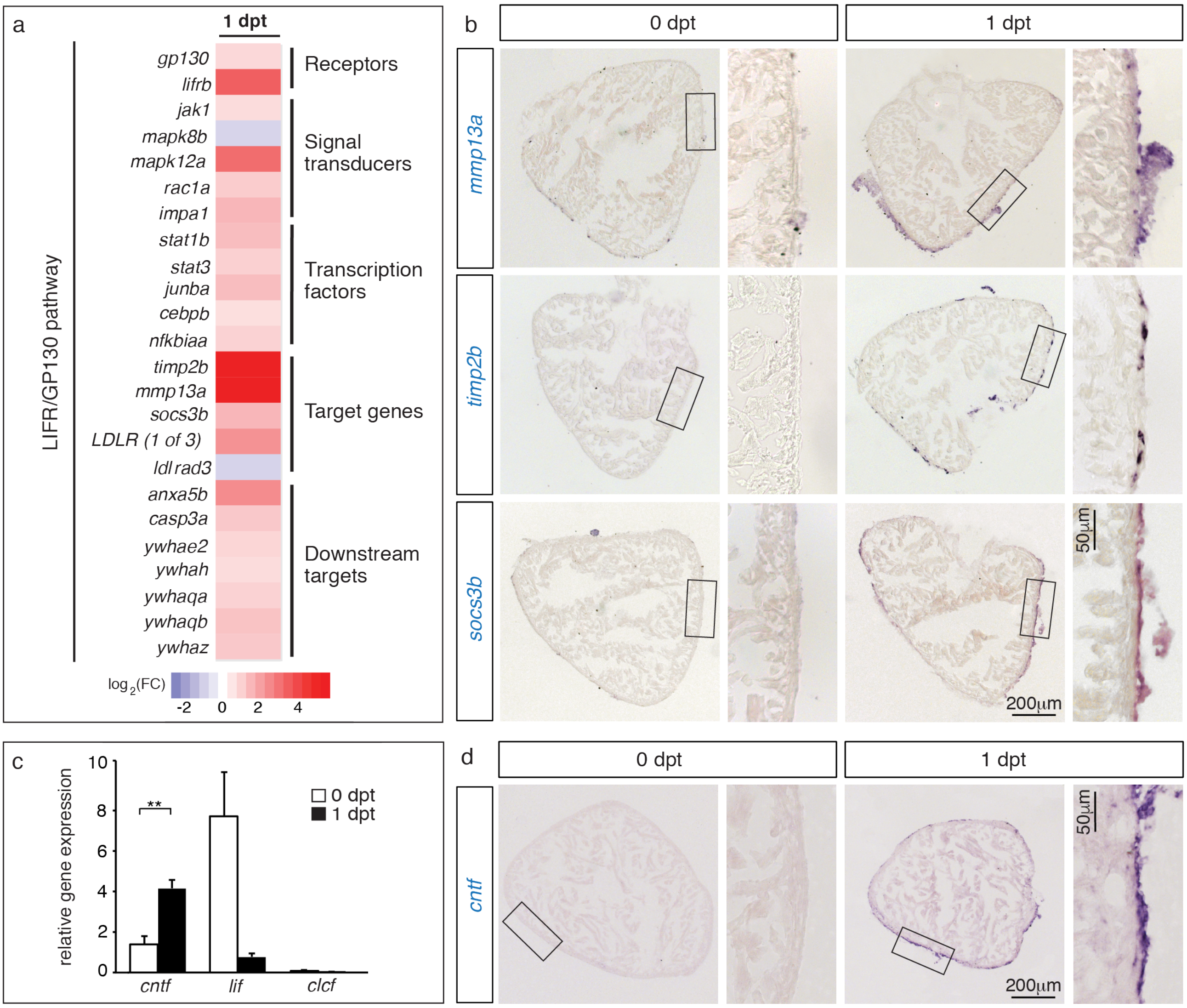

Fig. S2. Components of the LIFR/GP130 pathway are transcriptionally upregulated after preconditioning.

(a) Heat map representation of 20 differentially expressed genes at 1 dpt that contribute to the LIFR/gp130 pathway. Fold changes are represented in log2 scale of fold change (blue: log2 < 0; red: log2 > 0).

(b) *In-situ* hybridization of ventricular sections demonstrates upregulation of several components of the LIFR/GP130 pathway at 1 dpt. n ≥ 3 hearts.

(c) Detection of putative LIFR/gp130 ligands at 1 dpt by qRT-PCR. The normalized gene expression was calculated relative to *β-actin*. n ≥ 3 samples prepared of 10 ventricles. **P < 0.01 with student's t-test. (d) *In-situ* hybridization of ventricular sections demonstrates upregulation of *cntf* transcription at the periphery of the ventricle, at 1 dpt. n ≥ 3 hearts.

Supplemental Fig. S3

a

```
CNTF_Rattus norvegicus      mafaeqtpl-tlhrrdlcsrsiwlarkirsdltalmesyvkhqglnkninldsvdgvpva
CNTF_Danio rerio            --magrgtngrhglrgragtsaalarllhkeciqllksyrnseslps----spvegghli
                              :* :        *. .. *  *** ::.:   *::** : :.* .    . *:*  :

CNTF_Rattus norvegicus      std-rwsemteaerlqenlqayrtfqgmltklledqrvhftptegdfhqaihtlmlqvsa
CNTF_Danio rerio            sipplvpqlsdeekisilhaalkeclrlledvitredeefpdedseykrlrktvrdrlrh
                            *      :::: *::.    * :    :* .::  :  .*   :.::::  :*:  ::

CNTF_Rattus norvegicus      fayqleelmvlleqkipeneadgmpatvgdgglfekklwglkvlqelsqwtvrsihdlrv
CNTF_Danio rerio            llasteq-mmvdgqkf-eaevkeeldgqatggsfglkmwivrvfqelvhwtgqtsdalss
                            :  . *: *::  **: * *..      . ** *  *:* ::*:*** :** :: . *

CNTF_Rattus norvegicus      isshqmgisale-shygakdkqm
CNTF_Danio rerio            lpaekekapvsqrtrrgagklrk
                            : :.:    . : :: ** . :
```

b

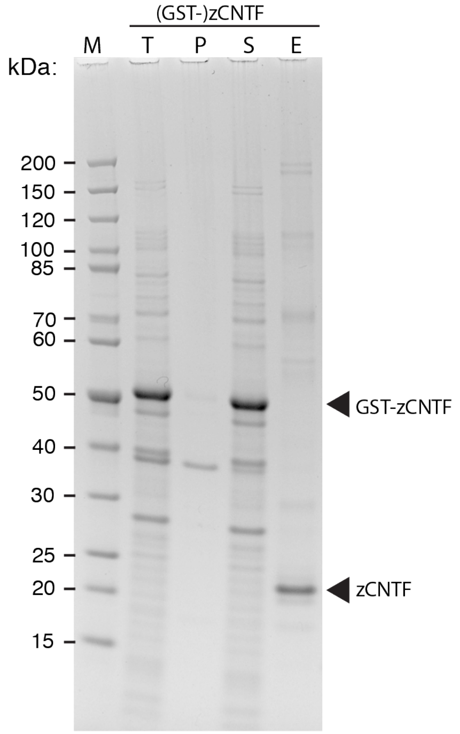

Fig. S3. Expression and purification of recombinant zebrafish CNTF protein.
(a) Sequence alignment between CNTF of *Rattus norvegicus* (NP_037298.1) and *Danio rerio* (NP_001139104.1) realised with Clustal Omega (EMBL-EBI website interface, see https://www.ebi.ac.uk/Tools/msa/clustalo/): Identity 18.71% - 38 identical (*) positions and 69 strongly (:) and weakly (.) similar positions.
(b) N-terminally GST-tagged zCNTF was expressed in *E. coli*, bound to glutathione (GSH) agarose beads, and eluted by thrombin cleavage. Proteins were separated by SDS-PAGE and revealed by Coomassie staining. T, total extract; P, pellet fraction (insoluble proteins); S, soluble extract (input for purification); E, eluate obtained by thrombin cleavage; M, molecular weight standard.
Proteins contained in the highlighted bands were identified by mass spectrometry: arrowheads indicate the bands corresponding to GST-zCNTF and eluted zCNTF (195 amino acids, expected molecular mass: 21.8 kDa).

Supplemental Fig. S4

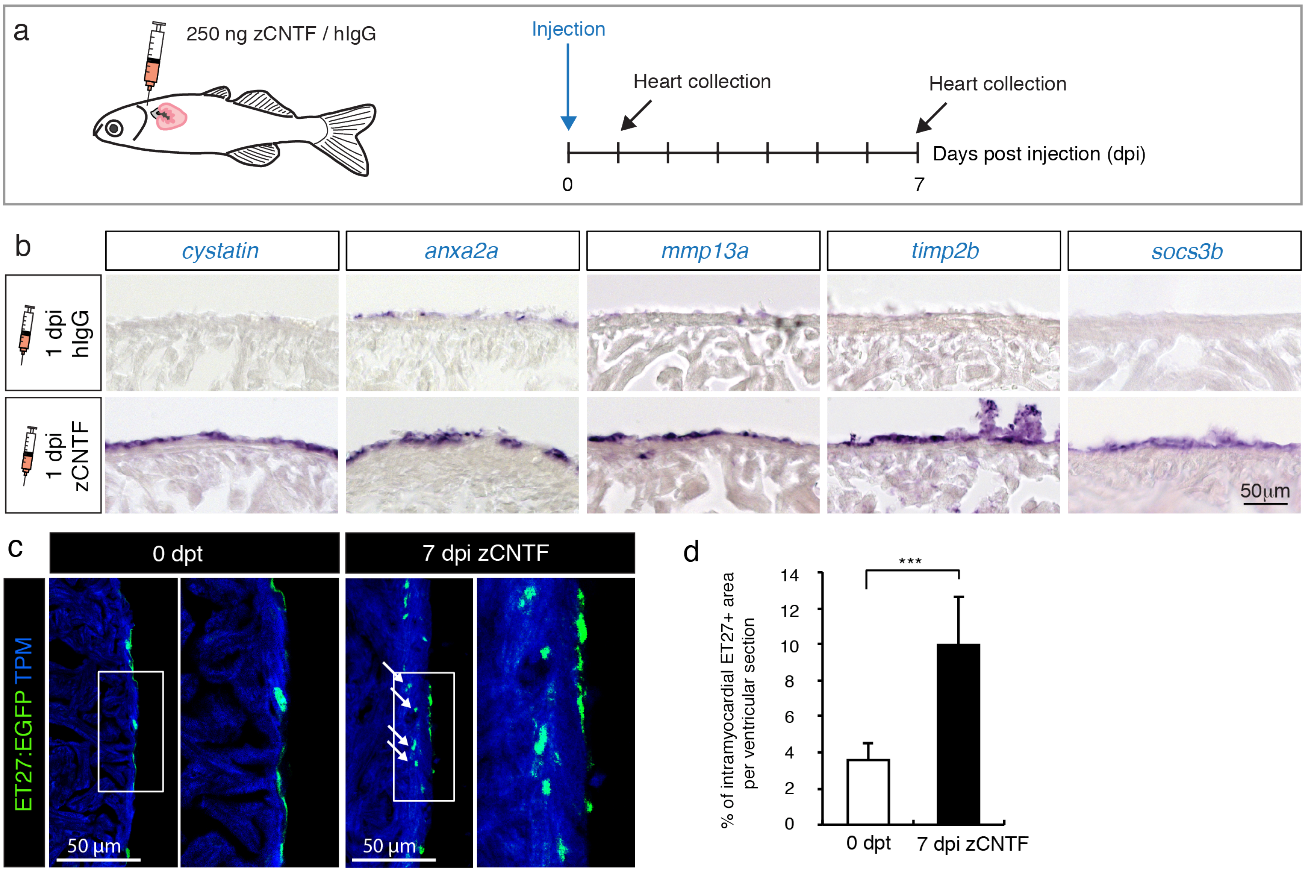

Fig. S4. Exogenous zCNTF induces expression of preconditioning genes and activates the epicardium.

(a) Experimental design to assess effects of a single pericardial injection of zCNTF on gene expression and the epicardium.

(b) *In-situ* hybridization of ventricular sections with different probes at 1 day post-injection (1 dpi) of control proteins (hIgG) and zCNTF. Several candidate genes identified in transcriptomic analysis are upregulated on the heart surface on the next day following zCNTF injection. n ≥ 3 hearts, 3 sections per heart each.

(c) Immunofluorescence staining of ventricular sections of the transgenic fish *ET27:EGFP* (green) with antibodies against cardiac Tropomyosin (TPM, blue). At 0 dpt, *ET27:EGFP+* cells are confined to the epicardium. At 7 dpi, *ET27:EGFP+* cells expand and infiltrate the myocardium (arrows). (d) Quantification of intramyocardial *ET27:EGFP+* area per ventricular section area. Superficial epicardial *ET27+* cells were not included in measurements. n ≥ 3 hearts, 3 sections per heart each. *** $P<0.001$with student’s t-test.

Supplemental Fig. S5

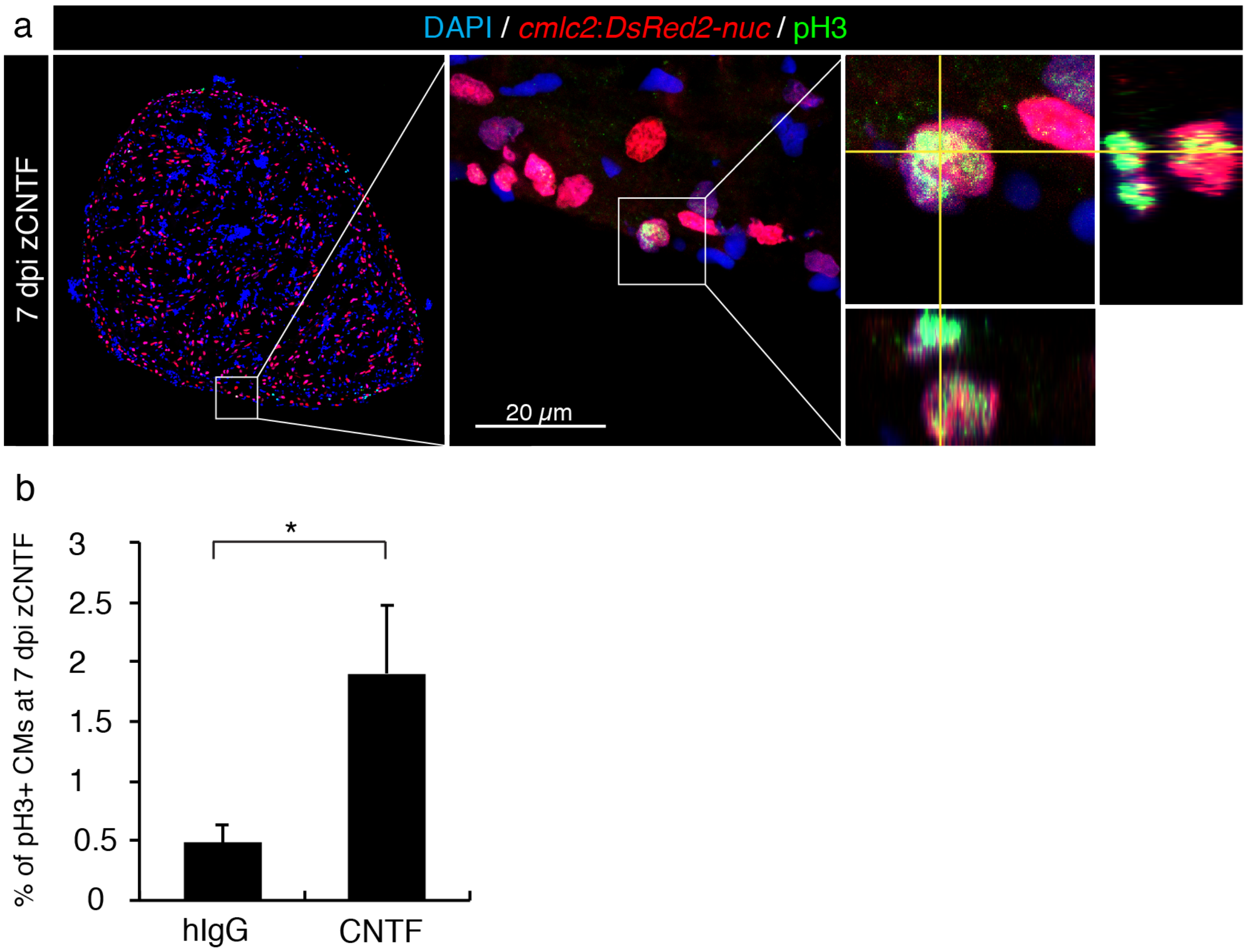

Fig. S5. Injected zCNTF increases mitosis in the uninjured zebrafish heart.
(a) Immunofluorescence staining of ventricular sections of the transgenic fish *cmlc2:DsRed2-nuc* (red) with antibodies against phospho-Histon H3 (pH3, green) of an intact heart at 7 dpi with zCNTF. The middle panel represents the higher magnification of the framed area in the left panel. The image on the right side shows a mitotic CM. Orthogonal projections along the yellow planes demonstrate a dividing CM.
(b) Quantification of pH3-positive CM at 7 dpi with hIgG and zCNTF. n ≥ 8 hearts, 5 sections per heart each. * $P<0.05$ with student's t-test.

Supplemental Fig. S6

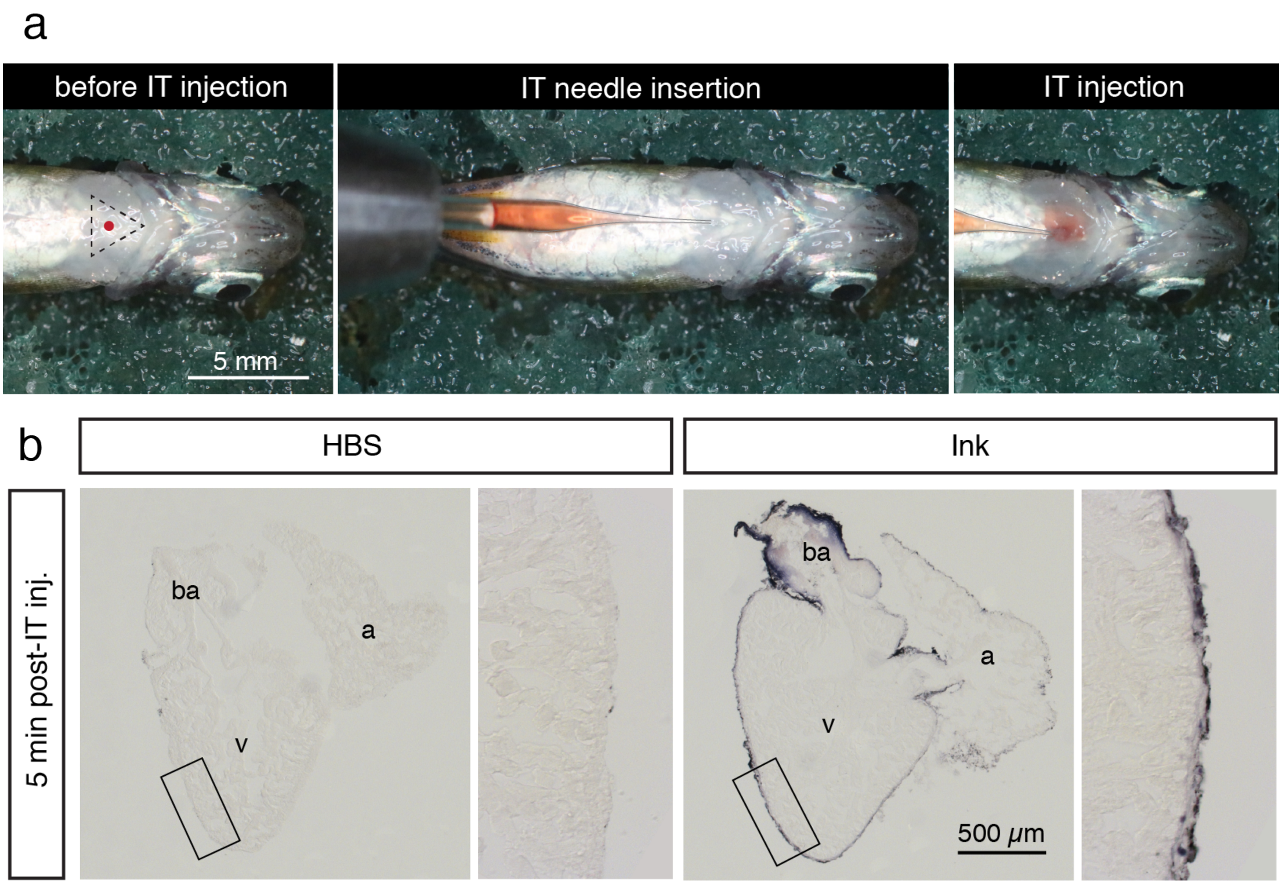

Fig. S6. Intrathoracic microinjection procedure.
(a) Photographs of the microinjection procedure under the stereomicroscope.
Before intrathoracic (IT) injection, an anesthetized fish was positioned with the ventral side up on a moist sponge. The tip of the pulled glass needle with a 2.5 µl solution was inserted under the skin of the thorax. The solution was slowly injected into the pericardial activity with a caution not to puncture the heart.
(b) Histological sections of the zebrafish heart for a validation of accuracy of the microinjection procedure. The fish were euthanized and IT-injection was performed using 2.5 µl Hank's buffer (HBS) or purple ink. At 5 min post injection, the hearts were collected, fixed and sectioned. Injected ink labeled the surface of the ventricle (v), atrium (a) and bulbus arteriosus (ba), suggesting efficient spreading of the injected solution throughout the pericardial cavity. No dye was observed inside the organ, demonstrating that the needle did not puncture the heart during injection.
